# Impact of Group Art Therapy Using Traditional Chinese Materials on Self-Efficacy and Social Function for Individuals Diagnosed With Schizophrenia

**DOI:** 10.3389/fpsyg.2020.571124

**Published:** 2021-01-20

**Authors:** Jie Tong, Wei Yu, Xiwang Fan, Xirong Sun, Jie Zhang, Jiechun Zhang, Tingting Zhang

**Affiliations:** Shanghai Pudong New Area Mental Health Center, Tongji University School of Medicine, Shanghai, China

**Keywords:** group art therapy, traditional Chinese materials, schizophrenia, social function, self-efficacy

## Abstract

The purpose of this study was to examine the effect of group art therapy using traditional Chinese materials on improving the self-efficacy and social function of individuals diagnosed with schizophrenia. In China, little research has been conducted on patients to measure the effectiveness of group art therapy, especially using traditional Chinese materials. To address this research gap, 104 individuals diagnosed with schizophrenia were tested in a group art therapy program that included 30 treatment sessions and used a wide variety of materials, including traditional Chinese materials, such as Chinese calligraphy, traditional Chinese painting, Chinese embroidery, and Chinese beads. The effect of art therapy was analyzed using the General Self-Efficacy Scale (GSES) and Scale of Social Skills for Psychiatric Inpatients (SSPI). This study demonstrates that group art therapy using traditional Chinese materials can improve self-efficacy and social function, reducing social and life function problems, and promote the recovery of individuals diagnosed with schizophrenia.

## Highlights

- The study uses traditional Chinese artistic practices and methods as a form of group art therapy, which is innovative in this field.
- Five themes are explored in the group art therapy intervention used in this study, each of which involves six stages. A variety of traditional Chinese mediums are used, including Chinese calligraphy, Chinese painting, Chinese embroidery, Chinese beading, and Chinese facial makeup.
- Group art therapy using traditional Chinese materials can improve self-efficacy and social function of individuals diagnosed with schizophrenia.

## Introduction

Schizophrenia is a group of diseases with unknown etiology and different mental symptoms involving many obstacles, such as those relating perception, thinking, emotion, and behavior, as well as uncoordinated mental activities (Esman, 2012). The natural course of the disease is generally prolonged and recurrent, which easily leads to a decline in social function. The lifetime prevalence of the disease is 1% worldwide (Gilmer et al., 2004). Patients with schizophrenia often have serious social function defects, which are closely related to self-efficacy and internal motivation (Vaskinn et al., 2015). Despite more than a century of research and major improvements in antipsychotic medication and psychological treatment, the majority of the burden of schizophrenia is unavoidable in light of current knowledge (Knapp, 2000; Nunes et al., 2018). Three-quarters of patients with schizophrenia experience recurrent and persistent symptoms with substantial impacts on their daily and social lives, and full remission is rare (Hasson-Ohayon et al., 2015). For the patients themselves, original social function and levels of self-concept and self-esteem are reduced, and there is a high medical expense cost. Thus, the quality of life of patients with schizophrenia is significantly lower than that of healthy individuals (Kostogianni and Andronikof, 2009).

In the 1980s, experiments showed that psychological intervention can improve the family relationships of individuals diagnosed with schizophrenia, improve social function, reduce the recurrence of disease, and renew people’s interest in psychotherapy (Galbusera et al., 2018). Psychological intervention can be divided into family intervention, cognitive behavioral psychotherapy, social skill training, and psychological education (Prochaska, 1979). Family intervention mainly reduces high levels of emotional expression in the family and enhances drug compliance (Cottrell et al., 2018). Cognitive behavioral psychotherapy mainly intervenes in patients’ coping strategies and behaviors as well as in their irrational beliefs related to symptoms (Stefan et al., 2014). The main purpose of social skills training is to improve interpersonal relationships by teaching social skills and creating a healthy interpersonal environment for patients (Kurtz et al., 2015). Psychological education allows patients to develop a comprehensive understanding of themselves through teaching and training and enhances the self-treatment ability of diseases and drugs (Ivey, 2014).

Art therapy originated from the psychotherapy movement of the 1940s, which was mainly influenced by two psychologists, Prof. Freud and Prof. Jung (Wilkinson and Chilton, 2013). It has always been an independent discipline, and art therapy was formalized in the early 1960s and introduced to China in the 1990s (Ting and Bing-Shuang, 2012). As stated by the American Art Therapy Association (AATA), the purpose of art therapy is “to provide non-verbal expression and communication opportunities by using art media, art creation process and the reflection of patients on the artistic works created, to realize the service of reflecting on personal development, ability, personality, interest, inner concerns, and conflicts.” (Potash et al., 2015). Prof. Ley, a psychologist, believes that “one cannot use the left hemisphere key to unlock the right hemisphere” (Sheikh and Shaffer, 1979). This view is based on the theory of the division of labor between two hemispheres of the brain. Language therapy is effective in correcting diseases caused by incorrect cognition or thinking but cannot deal with emotional disorders, traumatic experiences, and other problems with emotional distress as main symptoms (Maher et al., 2006). Through the process of artistic creation and the use of non-verbal tools, patients can identify suppressed feelings and conflicts from the subconscious, which can be expected to play a unique role in emotional, cognitive, and social function disorders (Jedidi et al., 2018). Unlike individual art therapy, group art therapy can reveal other people’s experiences and feelings from multiple perspectives and improve one’s ability to actively develop oneself (Deshmukh et al., 2018).

Relevant research and applied reports in the field of group art therapy are limited to date, and only painting therapy, music therapy, dance therapy, and exercise therapy have been explored in previous studies. Conway believes that painting therapy can improve the creativity of patients, promote the recovery of emotional and cognitive functions, improve social functions, and improve quality of life (Detrizio et al., 2013). A randomized controlled study involving 123 patients with schizophrenia showed a trend toward improved symptom scores among those randomized to music therapy, especially regarding the general symptoms of schizophrenia (Maratos, 2010). Prof. Biondo believes that dance therapy interventions have supported a decrease in psychological distress and positive and negative symptomatology for those with schizophrenia living in an inpatient psychiatric facility (Biondo and Bryl, 2020). Prof. Martin’s research shows that movement therapy has a positive effect on negative symptoms in patients with schizophrenia, matching or even surpassing the efficacy of conventional pharmacological and psychological treatment (Martin et al., 2016).

In China, services for individuals diagnosed with schizophrenia are still focused on drug treatment and closed management based on the medical model (Chen et al., 2011). Non-drug treatment and deinstitutionalization management have gradually drawn attention and will become the future development trend (Schiavo et al., 2017). Professionals have attempted to use a variety of psychotherapy techniques to help patients with mental disorders eliminate mental distress more quickly and explore more acceptable treatment methods for patients. Group art therapy is a form of psychotherapy with art applied with an intermediary and group approach, which has advantages in addressing emotional disorders (Shafir et al., 2020). However, Chinese patients have been influenced by Chinese traditional art education since childhood. Chinese patients are good at expressing their emotions by traditional artistic methods, such as brushwork, watercolor, and drama. It is difficult for them to understand the methods of art therapy schools, such as those employed in Europe and the United States, which reduces their interest in participating in such treatment and affects the expected impact of treatment. In anthropology, experimental manipulation of cultural or environmental conditions, such as language and esthetic, it is found that cultural differences can strongly inhibit individual experience. Cultural adaptation should be completely considered in the content of intervention. By integrating local cultural elements, the content of intervention is more acceptable to the local population (Mace and Jordan, 2011). Prof. Erikson, a famous American developmental psychologist and psychoanalyst, once said, “If you communicate with a person in a language that he understands, he will remember it in his mind; if you use their experience to communicate with him, he will keep it in mind” (Brown and Lowis, 2003). Thus, in this study, we use traditional Chinese art forms that Chinese patients are familiar with, esthetic experiences they have had, and tools they have mastered, such as Chinese calligraphy, Peking opera mask making, and embroidery, in group art therapy courses. When works are presented and shared, patients can experience more resonance, relieve negative depressive emotions, show reduced resistance to treatment, and identify more of their inner experiences. This study explores the effects of group art therapies using traditional Chinese materials on the self-efficacy and social function of individuals diagnosed with schizophrenia.

## Materials and Methods

### Participants

The sample size of the program is calculated based on the formula ${\text{N}}_{\text{sample}}={\left[\frac{{\text{Z}}_{a/2}\,\sqrt{\pi_{0}\,\left(1-\pi \right)}+Z_{\beta }\,\sqrt{\pi \,\left(1-\pi \right)}}{\pi -\pi_{0}}\right]}^{2}$ (Farrington and Manning, 2010). The significance level is 0.05, and the expected effective rate is 80%. Thirty cases were needed for both the experimental group and the control group, and 20% of the lost samples were added (those not discharged automatically according to the discharge standards or that could not be completed for other special reasons) with 50 cases included in the experimental group and 50 cases included in the control group.

A total of 110 in-patients with schizophrenia living in our hospital from July 2017 to July 2018 were selected. The following inclusion criteria were employed: (1) patients meeting the DSM-5 (Roehr, 2013) diagnostic criteria; (2) 18 years old ≥ age ≤ 60 years old; (3) continuous hospitalization ≥6 months; (4) types and doses of antipsychotic drugs used are basically unchanged; (5) certain visual and auditory resolution without understanding disorders; (6) capacity to independently complete the self-test scale; (7) education above primary school; (8) capacity to participate in sports activities; and (9) PANSS total score ≤60. The following exclusion criteria were employed: (1) discharge within half a year of observation; (2) combination of three or more drugs used during observation; (3) inability to continue observation due to serious physical disease; and (4) dementia/developmental delay diagnosis with behavioral disorder. The participants were divided into an experimental group and a control group using a random number table, and each group included 55 cases. Two cases in the experimental group and four cases in the control group were removed due to discharge, leaving 104 cases in the final study.

### Group Art Therapy Intervention

The patients in the experimental group participated in group art therapy using traditional Chinese materials. The patients in the control group were also given common materials at the same time for free creation but were not given art tasks or intervention procedures. The experimental and control groups were divided into five groups with approximately 10 patients in each group. Each group was treated twice a week for 90 min for a total of 30 times in 15 weeks. The experimental group was treated by the same art therapist. The art therapist has a bachelor’s degree in Oriental Art and a master’s degree in psychology and is a licensed Chinese art therapist. The staff was responsible for distributing common materials to the control group without art guidance or analysis. The two groups convened in different treatment rooms at the same time without interference.

**FIGURE 1:**
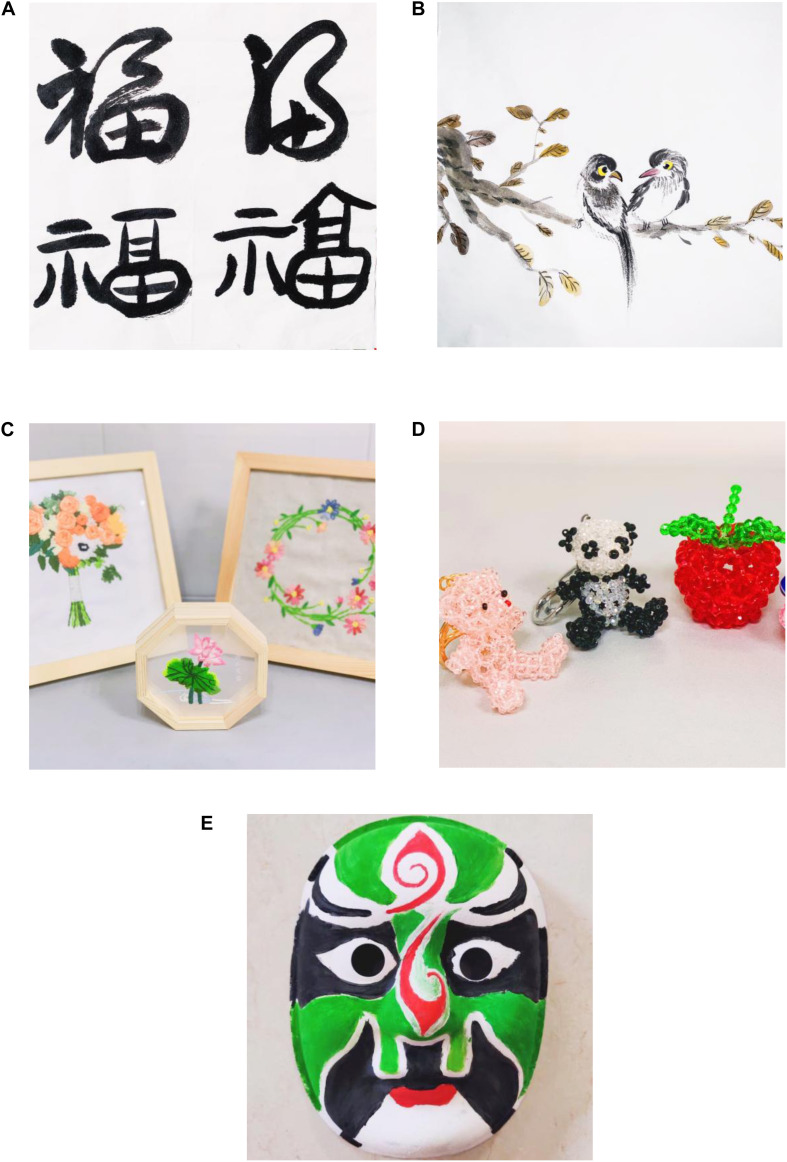
**(A)** Chinese calligraphy; **(B)** traditional Chinese painting; **(C)** Chinese embroidery; **(D)** Chinese beading; and **(E)** Chinese Peking Opera mask.

Based on the structured group art intervention model developed by Luzzatto and Gabriel (2000) and using traditional Chinese materials, a group art therapy course was designed according to the actual situations of the patients with schizophrenia (Teglbjaerg, 2011). The course is divided into five art themes: Chinese calligraphy (Figure 1A), Chinese painting (Figure 1B), Chinese embroidery (Figure 1C), Chinese beading (Figure 1D), and Chinese Peking Opera facial makeup artistry (Figure 1E). Each theme is explored over six stages. For example, the theme of Chinese calligraphy covers free style, basic methods, favorite words, happiest memories, group work, and the sharing of successful experiences (see Table 1).

**TABLE 1:** Group art therapy task.

**Course of treatment**	**Theme**				
	
	**Chinese calligraphy**	**Traditional Chinese painting**	**Chinese embroidery**	**Chinese beads**	**Chinese Peking Opera mask**
1	Free style	Free style	Free style	Free style	Free style
2	Provide basic guidance	Provide point foundation	Provide basic guidance	Provide basic guidance	Provide basic guidance
3	Favorite words or sentences	Provide line foundation	Theme 1: Hesitation or frustration	Theme 1: My past	Theme 1: Fear or worry
4	Happiest memory	Provide graphic foundation	Theme 2: Family	Theme 2: My present	Theme 2: Peace or comfort
5	Group work	Provide color foundation	Group work	Group work	Our faces
6	Share successful experiences	Share successful experiences	Share successful experiences	Our ideal space	Life after recovery

(1) Free style. Art therapists distribute traditional Chinese art materials and allow the patients to create freely. During the period of creation, art therapists do not provide any guidance. At the end of the creation period, each participant introduces the meaning of his or her artwork and allows the other participants to share their views. The art therapists do not comment on the artworks and only encourage or affirm the patients’ courage to participate. The goal is to improve the participation and enthusiasm of the patients in the treatment and increase their sense of initiative.
(2) Providing basic guidance. First, the art therapists explain the origins and development of traditional Chinese art and demonstrate artistic methods on site. Then, the participants copy these methods or create freely. During the period of creation, the art therapists provide basic guidance. Afterward, each participant shares his or her experiences with the exercise. The art therapists review each artwork created. The goal is for the patients to describe their current psychological states and improve their self-confidence.
(3) Favorite words or sentences. First, each participant writes his or her favorite words or sentences down. Then, the participants create art using traditional Chinese art materials centering on these words or sentences. Afterward, patients individually present their favorite words or sentences and artworks and share why they have selected the presented words or sentences. The art therapists analyze the relationships between the selected words or sentences and the artwork. The goal is to encourage the patients to express their inner experiences, experience positive emotions, and address negative emotions.
(4) Happiest memories. First, each participant writes down one of his or her best memories, which can involve an event, a person, or an object. Then, the participants create art using traditional Chinese art materials. The artwork can express a participant’s happiest memory or that of someone else. Afterward, the patients individually describe their selected memories and artworks. Finally, art therapists analyze the artwork and the patients’ past experiences. The goal is to express past experiences through artwork and strengthen patients’ self-image through the expressions of characters, positive environments, or moods that have influenced them.
(5) Group work. First, 10 participants are randomly divided into two groups with five in each group. Each group discusses and selects a theme and then develops artistic creations according to this theme. Members of each group work together to complete this relatively complex artistic task. After creation, each group selects a representative to elaborate on the significance of the artworks or on interesting events that occurred in the process of artistic creation. The goal is to have the patients establish positive interpersonal relationships and improve their self-efficacy.
(6) Sharing successful experiences. Each participant shares his or her artwork created on the selected theme and his or her experience with the group art therapy session and expectations of life. Other participants are invited to resonate with what is shared. Finally, art therapists summarize the theme selected and again describe the purpose of group art therapy. The goal is to allow the patients to face themselves, live in the present, conform to current circumstances, and regain a sense of hope that they will one day return to social life.

Each group art therapy session can be roughly divided into the following steps. (1) Distribute art therapy tools and materials. Let the patients become familiar with the therapy tools and materials and learn to express their feelings using artistic methods. (2) Clarify the purpose and task of group art therapy. With the guidance of art therapists, patients are asked to draw from image-focused information stored in the right brain, to learn this new language with a positive and optimistic attitude, and to express internal changes through artistic creation. (3) Assist with the task. In the process of artistic creation, art therapists assist patients in completing artwork, help them identify the techniques that they like, and help them use these techniques to produce meaningful art according to their own wishes. (4) Review and analyze the artwork. Let the patient explain the meaning of the artwork and express subjective experiences and personal stories. From the artwork created, art therapists can understand the problems and psychological experiences of the patients and find opportunities for intervention. (5) End the intervention process. Identify problems, reveal the essence of these problems, and create solutions.

### Measure

The purpose of this study was to examine how effectively group art therapy works when patients with schizophrenia use traditional Chinese materials to enhance their self-efficacy and improve social function. The study applied both qualitative and quantitative assessments of patients’ responses to effectively draw out the more nuanced results of group art therapy using traditional Chinese materials in supporting their psychological conditions (Kim et al., 2016).

The General Self-Efficacy Scale (GSES) was used to evaluate the self-efficacy of the patients. The GSES was compiled by Prof. Ralk Schwarzer in 1981, a clinical psychologist at the Free University of Berlin, Germany (Schwarzer and Born, 1997). The Chinese version of the GSES was introduced to China by Prof. Jianxin Zhang in 1995 (Zhang and Schwarzer, 1995). The scale measures the following 10 items: (1) If I try my best, I can always solve problems; (2) I can still get what I want even though others are against me; (3) It is easy for me to stick to my ideals and achieve my goals; (4) I have confidence that I can deal with unexpected events effectively; (5) With my intelligence, I can cope with unexpected situations; (6) When I make the necessary efforts, I can solve most problems; (7) I can face difficulties calmly because I trust my ability to deal with problems; (8) When facing a difficult problem, I can usually find several solutions; (9) When I experience challenges, I usually think of ways to deal with them; and (10) No matter what happens to me, I can handle it easily. Using Likert’s 4-grade scoring method, “totally incorrect” is scored with one point, “somewhat correct” is scored with two points, “mostly correct” is scored with three points, and “completely correct” is scored with four points. A total score ranges from 10 to 40 points. The higher the total score, the higher the degree of self-efficacy. The GSES has good reliability and validity and is suitable for the quantitative evaluation of the self-efficacy of schizophrenia patients (Chen et al., 2001). The scale was completed by patients themselves. The Cronbach’s α coefficient of the scale was 0.831.

The Scale of Social Skills for Psychiatric Inpatients (SSPI) was used to evaluate the social functions of the patients. The SSPI was compiled by Prof. Chaodang Zhou in 2003 (Chaodang and Shuchun, 2004). The SSPI includes 12 items in total, including factor I: daily living ability; factor II: mobility and communication; and factor III: social activity skills. Using Likert’s 5-grade scoring method, “a lack of this function” is scored with zero points; “the need to expend considerable energy to complete a project” is scored with one point; “the existence of this function, but only under supervision” is scored with two points; “the ability to complete a project independently, but with little enthusiasm or initiative” is scored with three points; and “the ability to perform well consistently” is scored with four points. The total score ranges from 0 to 408 points. The higher the total score, the stronger the social function. The SSPI has good reliability and validity and is suitable for the quantitative evaluation of the social function of patients with schizophrenia (Bhola et al., 2016). The scale was evaluated by trained professionals. The Cronbach’s α coefficient of the scale was 0.871.

The GSES and SSPI were evaluated at baseline and 15 weeks after intervention. The GSES was completed by patients themselves. The SSPI was assessed by five psychiatrists who received unified training. Kappa was set to 0.83 for consistency (*p* > 0.05).

### Data Management and Analysis

Data were analyzed using SPSS 20.0. The demographic characteristics of the two groups were analyzed using independent sample and chi-square tests. The baseline GSES and SSPI scores of the two groups were analyzed using independent sample t-tests. The GSES and SSPI scores of the two groups before and after intervention were analyzed using paired t-tests within groups.

## Results

### Sample Profile

There were no significant differences in sex, age, course of disease, or education level between the two groups (see Table 2).

**TABLE 2:** Demographic characteristics.

**Variable**	**Experimental group**	**Control group**	***p*/*t***	***p***
Sex (male/female)	28/25	25/26	0.15	0.70
Age (year)	43.68 ± 7.25	46.06 ± 8.26	1.56	0.12
Course of disease (year)	12.32 ± 5.15	13.69 ± 4.44	1.45	0.15
Education level (year)	10.75 ± 2.17	10.35 ± 2.50	−0.88	0.38

*Data are presented as means.*

### General Self-Efficacy Analysis

The experimental group (EG) mean General Self-Efficacy Scale (GSES) scores for before and after the experiment are significantly different. The mean score of the EG before the experiment is 19.68, whereas the post-experiment score is 41.08 (*p* = 0.007). On the other hand, the scores of control group (CG) do not significantly differ between the pre- and post-experiment results. The mean of the CG is 19.27 before the experiment and 20.31 after the experiment (*p* = 0.907, see Table 3).

**TABLE 3:** General Self-Efficacy Scale (GSES) scores of the experimental and control groups.

**General Self-Efficacy Scale**	**Pre**		**Post**		***t***	***p***
	***n***	**M(SD)**	***n***	**M(SD)**		
1	53	19.68 (4.71)	53	41.08 (6.10)	13.112	0.007*
2	51	19.27 (7.05)	51	20.31 (5.02)	0.001	0.907

Experimental group-1; control group-2. *p < 0.01.

### Analysis of Psychiatric Inpatients’ Social Skills

As Table 4 shows, group art therapy was effective across all three subcategories and for the total score of the Scale of Social Skills for Psychiatric Inpatients (SSPI). The pretest and posttest figures are significantly different (*p* < 0.01) for the EG, whereas none of the figures are significantly different for the CG. The mean daily life ability score for group art therapy before the experiment is 8.13, and this figure increases to 3.81 after the experiment, which is significantly different (*p* = 0.005). On the other hand, the mean score is 8.14 before the test and increases to 8.87 after the intervention, and the values minimally change for the CG. The mean mobility and communication score before the experiment is 10.68, and this figure increases to 8.42 after the experiment, which is significantly different (*p* = 0.009). However, the mean score is 10.12 before the test and increases to 10.41 after the intervention; minimal changes occurred in the CG. For social activity skills, the mean score for the EG is 6.26, and this figure increases to 8.17 after the experiment, which is statistically significant (*p* = 0.006). A slight and insignificant difference in the CG occurred with a mean score of 5.61 before the test and 5.53 after the test. Regarding the total SSPI score, the mean is 25.08 before the experiment, and the figure increases to 45.48 after the experiment for the EG, which is statistically significant (*p* = 0.008; see Table 4).

**TABLE 4:** Scale of Social Skills for Psychiatric Inpatients (SSPI) scores of the experimental and control groups.

**Scale of Social Skills**		**Pre**		**Post**		***t***	***p***
**for Psychiatric Inpatients**		**Mean**	**SD**	**Mean**	**SD**		
Daily life abilities	1	8.13	2.25	11.94	2.13	6.951	0.005*
	2	8.14	1.10	8.87	2.47	0.003	0.991
Mobility and communication	1	10.68	3.01	19.10	3.63	12.105	0.009*
	2	10.12	1.82	10.41	3.36	0.004	0.253
Social activity skills	1	6.26	2.62	14.43	3.40	13.300	0.006*
	2	5.61	2.68	5.53	2.92	0.009	0.202
SSPI total score	1	25.08	6.93	45.48	8.131 (5.02)	12.317	0.008*
	2	23.86	4.37	24.80	8.01	0.001	0.281

Experimental group-1; control group-2. *p < 0.01.

## Discussion

This study examined how group art therapy using traditional Chinese materials affects the self-efficacy and social function of individuals diagnosed with schizophrenia. The subjects were randomly assigned to control differences between the experimental and control groups before intervention. The statistical analysis of psychiatric evaluation and pretest results revealed no statistical differences between the two groups, indicating that the randomized treatment achieved the goal of controlling differences between the experimental and control groups.

Self-efficacy is defined as a subjective evaluation of an individual’s self-belief in completing an activity to adapt to the environment and of the possible mastery of the activity (Bandura, 1977). Crawford found the evaluation of the three dimensions of physical function, psychological function, and social function in a low self-efficacy group to be significantly lower than that in a high self-efficacy group, indicating that the quality of life in patients with schizophrenia with low self-efficacy is significantly lower than that in patients with high self-efficacy (Crawford et al., 2010). The results of our study show that the GSES scores of the experimental groups increased after group art therapy using traditional Chinese materials, and a significant difference was noted between the two groups. The self-efficacy of patients with schizophrenia improved. Before intervention, coping styles, such as retreat, fantasizing, self-blame, problem solving, and help seeking, were mainly used in a negative manner (Dresp-Langley et al., 2017). After the intervention, the coping styles were used in a positive manner. In expressing difficulties and pressures to a psychotherapist instead of adopting irrational coping styles, such as social withdrawal and fantasizing, and with the formation of successful experiences through the use of reasonable coping styles, a patient’s levels of self-efficacy can be improved (Corcoran, 2015). The patient can subsequently recognize the objective features of themselves and actively cooperate with the psychotherapist to complete various therapies (Carr, 2014). When facing difficult challenges, the patient can ask the psychotherapist or family members for help instead of complaining or feeling inferior (Chilton and Scotti, 2014). This finding demonstrates that group art therapy using traditional Chinese materials can improve patients’ self-efficacy, which is consistent with the results of international research (Kim, 2013^∗^).

Currently, the treatment of schizophrenia is still dominated by drug treatment worldwide (Meltzer, 2017). However, drug treatment does not easily improve the social functions damaged by the disease (Albert and Shih-Jen, 2017). We found that SSPI scale, daily life ability, mobility and communication, and social activity skill factor scores of our experimental group increased after group art therapy using traditional Chinese materials, and significant differences were found between the two groups. This finding demonstrates that the proposed intervention can improve the social function of patients, which is consistent with the results of international research (Huet and Holttum, 2016). In addition, the patients’ limited daily living ability levels were restored, resulting in them paying attention to personal hygiene, maintaining their appearance, making their beds, and following a balanced diet (Schrank et al., 2016). An improvement in the communication function is reflected by a harmonious relationship with the environment and with other people and resulted in increased enthusiasm among the patients and a willingness to share their feelings and show their talents (Naeem et al., 2006). Patients can actively access new information from multimedia, participate in discussions, and have a positive attitude. They can improve their self-management abilities and engage in some activities or work tasks. The patients feel valued, and their self-confidence is enhanced. These changes effectively address experiences with communication barriers, loneliness, and withdrawal.

## Conclusion

This study shows that group art therapy using traditional Chinese materials can improve self-efficacy and social function, reducing social and life function problems, and promote the recovery of individuals diagnosed with schizophrenia. The proposed intervention involving traditional Chinese art making is simple, easy to implement, and easy to accept and master. The approach can improve a patient’s confidence in dealing with difficulties using various skills, promote psychosocial rehabilitation, and enable a faster return to society.

## Limitations and Recommendations for Future Studies

The present study has several limitations that provide directions for future research. First, this study is limited by linguistic issues given that all data were collected in Chinese and were translated into English by the researcher. This process might have resulted in the participants’ statements being conveyed differently from what they had originally meant. Second, to explore the effect of group art therapy using traditional Chinese materials on individuals diagnosed with schizophrenia, samples using common materials were not included. Further work must include research samples using common materials to explore the effects of group art therapy on those from different cultural backgrounds. Third, this study is limited to individuals diagnosed with schizophrenia and does not assess other diseases. More diseases should be included in future studies to explore the effects of group art therapy on different diseases. Fourth, given the lack of previous research on group art therapy, this study is not informed by a large set of past data and literature references.

## Data Availability Statement

The raw data supporting the conclusions of this article will be made available by the authors, without undue reservation.

## Ethics Statement

The studies involving human participants were reviewed and approved by the Ethics Committee of Shanghai Pudong New Area Mental Health Center and Tongji University Mental Health Center (No: 201611). The patients/participants provided their written informed consent to participate in this study.

## Author Contributions

JT and WY: conceptualization. JT, WY, and XF: methodology, analysis and interpretation, and writing—review and editing. JT and XS: data analysis and writing—original draft preparation. JT: supervision. XS: project administration. JZ, JcZ, and TZ: sample collection. All authors approved the submitted version of the manuscript.

## Conflict of Interest

The authors declare that the research was conducted in the absence of any commercial or financial relationships that could be construed as a potential conflict of interest.

## References

[B1] lbert Y. Shih-Jen T. (2017). New targets for schizophrenia treatment beyond the dopamine hypothesis. *Int. J. Mol. Ences* 18:1689. 10.3390/ijms18081689 28771182 PMC5578079

[B2] Bandura A. (1977). Self-efficacy: toward a unifying theory of behavioral change. *Psychol. Rev.* 84 191–215. 10.1037/0033-295X.84.2.191 847061

[B3] Bhola P. Basavarajappa C. Guruprasad D. Hegde G. Khanam F. Thirthalli J. (2016). Development of a social skills assessment screening scale for psychiatric rehabilitation settings: a pilot study. *Indian J. Psychol Med.* 38 395–403. 10.4103/0253-7176.191392 27833220 PMC5052950

[B4] Biondo J. Bryl K. (2020). S190. WHEN WORDS AREN’T ENOUGH: DANCE/MOVEMENT THERAPY AND SCHIZOPHRENIA. *Schizophr. Bull.* 46 S110–S111. 10.1093/schbul/sbaa031.256 20421336

[B5] Brown C. Lowis M. J. (2003). Psychosocial development in the elderly: an investigation into Erikson’s ninth stage. *J. Aging Stud.* 17 415–426. 10.1016/S0890-4065(03)00061-6

[B6] Carr S. M. D. (2014). Revisioning self-identity: the role of portraits, neuroscience and the art therapist’s ‘third hand’. *Int. J. Art Therapy* 19 54–70. 10.1080/17454832.2014.906476

[B7] Chaodang Z. Shuchun J. (2004). A self-designed scale for social function in in-patients with psychosis (SSFPI):preliminary test of reliability and validity. *Sichuan Ment. Health* 17 144–146. 10.3969/j.issn.1007-3256.2004.03.006

[B8] Chen G. Gully S. M. Eden D. (2001). Validation of a new general self-efficacy scale. *Organ. Res. Methods* 4 62–83. 10.1177/109442810141004

[B9] Chen X. S. Xu Y. F. Tang Y. X. Wang Y. Zhang M. D. Lou F. Y. (2011). [Preliminary study on variations and neural generators of error-related negativity in first episode schizophrenics]. *Zhonghua Yi Xue Za Zhi* 91 3040–3043. 10.1007/s12264-011-1035-3 22333055

[B10] Chilton G. Scotti V. (2014). Snipping, gluing, writing: the properties of collage as an arts-based research practice in art therapy. *Art Therapy* 31 163–171. 10.1080/07421656.2015.963484

[B11] Corcoran K. D. Jr. (2015). *Hitting the Pause Button: An Investigation of Meditation, Mindfulness, and Empathic Communication.* Dissertations, Gradworks, Atlanta, GA.

[B12] Cottrell D. J. Wright-Hughes A. Collinson M. Boston P. Farrin A. J. (2018). Effectiveness and cost-effectiveness of systemic family therapy compared with treatment as usual for young people after self-harm: a pragmatic randomised controlled trial. *Lancet Psychiatry* 18 203–229. 10.1016/S2215-0366(18)30058-0 PMC5835764 29449180

[B13] Crawford M. J. Killaspy H. Kalaitzaki E. Barrett B. Waller D. (2010). The MATISSE study: a randomised trial of group art therapy for people with schizophrenia. *BMC Psychiatry* 10:65. 10.1186/1471-244X-10-65 20799930 PMC2940860

[B14] Deshmukh S. R. Holmes J. Cardno A. (2018). Art therapy for people with dementia. *Cochrane Database. Syst. Rev.* 9:Cd011073. 10.1002/14651858.CD011073.pub2 30215847 PMC6513479

[B15] Detrizio D. Emmerling D. Kowitt S. Mershon C. H. (2013). *Burma Art Therapy Project: Designing an Outcome Evaluation of an Art Therapy Program for Refugee Students from Burma in Chapel Hill-Carrboro City Schools.* Chapel Hill, CA: University of North Carolina at Chapel Hill.

[B16] Dresp-Langley B. Grossberg S. Reeves A. (2017). Editorial: perceptual grouping—the state of the art. *Front. Psychol.* 8:67. 10.3389/fpsyg.2017.00067 28194124 PMC5277007

[B17] Esman A. H. (2012). A history of adolescent psychiatry. *J. Nervous Ment. Dis.* 200 1058–1060. 10.1097/NMD.0b013e318275d25e 23197120

[B18] Farrington C. P. Manning G. (2010). Test statistics and sample size formulae for comparative binomial trials with null hypothesis of non-zero risk difference or non-unity relative risk. *Stat. Med.* 9 1447–1454. 10.1002/sim.4780091208 2281232

[B19] Galbusera L. Finn M. T. Fuchs T. (2018). Interactional synchrony and negative symptoms: an outcome study of body-oriented psychotherapy for schizophrenia. *Psychother. Res.* 28 457–469. 10.1080/10503307.2016.1216624 27687477

[B20] Gilmer T. P. Dolder C. R. Lacro J. P. Folsom D. P. Lindamer L. Garcia P. (2004). Adherence to treatment with antipsychotic medication and health care costs among Medicaid beneficiaries with schizophrenia. *Am. J. Psychiatry* 161 692–699. 10.1176/appi.ajp.161.4.692 15056516

[B21] Hasson-Ohayon I. Avidan-Msika M. Mashiach-Eizenberg M. Kravetz S. Rozencwaig S. Shalev H. (2015). Metacognitive and social cognition approaches to understanding the impact of schizophrenia on social quality of life. *Schizophr. Res.* 161 386–391. 10.1016/j.schres.2014.11.008 25499045

[B22] Huet V. Holttum S. (2016). Art therapy-based groups for work-related stress with staff in health and social care: an exploratory study. *Arts Psychother.* 50 46–57. 10.1016/j.aip.2016.06.003

[B23] Ivey A. E. (2014). Cultural expertise: toward systematic outcome criteria in counseling and psychological education. *J. Counsel. Dev.* 55 296–302. 10.1002/j.2164-4918.1977.tb04992.x

[B24] Jedidi H. Laverdeur C. Depierreux-Lahaye F. Beckers A. (2018). [A brief history of syphilis. The disease through the art and the artist]. *Rev. Med. Liege* 73 363–369. 30113775

[B25] Kim H. K. Kim K. M. Nomura S. (2016). The effect of group art therapy on older Korean adults with neurocognitive disorders. *Arts Psychother.* 47 48–54. 10.1016/j.aip.2015.11.002

[B26] Kim S. K. (2013^∗^). A randomized, controlled study of the effects of art therapy on older Korean-Americans’ healthy aging. *Arts Psychother.* 40 158–164. 10.1016/j.aip.2012.11.002

[B27] Knapp M. (2000). Schizophrenia costs and treatment cost-effectiveness. *Acta Psychiatr. Scand. Suppl.* 2000 15–18. 10.1046/j.1467-0658.2001.00137.x-i1 11261634

[B28] Kostogianni N. Andronikof A. (2009). [Self-esteem, self-centeredness and social-emotional adjustment of gifted children and adolescents]. *Encephale* 35 417–422. 10.1016/j.encep.2008.10.006 19853713

[B29] Kurtz M. M. Mueser K. T. Thime W. R. Corbera S. Wexler B. E. (2015). Social skills training and computer-assisted cognitive remediation in schizophrenia. *Schizophr. Res.* 162 35–41. 10.1016/j.schres.2015.01.020 25640526 PMC5146951

[B30] Luzzatto P. Gabriel B. (2000). The creative journey: a model for short-term group art therapy with posttreatment cancer patients. *Art Therapy* 17 265–269. 10.1080/07421656.2000.10129764

[B31] Mace R. Jordan F. M. (2011). Macro-evolutionary studies of cultural diversity: a review of empirical studies of cultural transmission and cultural adaptation. *Philos. Trans. R. Soc. Lond. B Biol.* 366 402–411. 10.1098/rstb.2010.0238 21199844 PMC3013475

[B32] Maher L. M. Kendall D. Swearengin J. A. Rodriguez A. Leon S. A. Pingel K. (2006). A pilot study of use-dependent learning in the context of constraint induced language therapy. *J. Int. Neuropsychol. Soc.* 12 843–852. 10.1017/S1355617706061029 17064447

[B33] Maratos A. (2010). W03-01 - A randomised controlled trial of co-improvisational music therapy for inpatients with schizophrenia, London/UK. *Eur. Psychiatry* 25 132–132. 10.1016/S0924-9338(10)70132-2

[B34] Martin L. A. L. Koch S. C. Dusan H. Thomas F. (2016). Overcoming disembodiment: the effect of movement therapy on negative symptoms in schizophrenia—A multicenter randomized controlled trial. *Front. Psychol.* 7:483. 10.3389/fpsyg.2016.00483 27064347 PMC4815039

[B35] Meltzer H. Y. (2017). New trends in the treatment of schizophrenia. *CNS Neurol. Disord. Drug Targets* 16 900–906. 10.2174/1871527316666170728165355 28758583

[B36] Naeem F. Kingdon D. Turkington D. (2006). Cognitive behaviour therapy for schizophrenia: relationship between anxiety symptoms and therapy. *Psychol. Psychother.* 79 153–164. 10.1348/147608305X91538 16774715

[B37] Nunes M. V. Adelino M. P. M. Ajub E. Quarantini L. C. Lacerda A. L. T. (2018). Efficacy of esketamine in the treatment of negative symptoms in schizophrenia - A case series. *Schizophr. Res.* 202 394–396. 10.1016/j.schres.2018.06.034 29935883

[B38] Potash J. S. Doby-Copeland C. Stepney S. A. Washington B. N. Vance L. D. Short G. M. (2015). Advancing multicultural and diversity competence in art therapy: American art therapy association multicultural committee 1990–2015. *Art Therapy* 32 146–150. 10.1080/07421656.2015.1060837

[B39] Prochaska J. O. (1979). Systems of psychotherapy: a transtheoretical analysis. *J. Comput. Theor. Nanosci.* 12 2732–2739.

[B40] Roehr B. (2013). American psychiatric association explains DSM-5. *Bmj* 346:f3591. 10.1136/bmj.f3591 23744600

[B41] Schiavo C. Tate A. Penna M. Stampella L. Grendas L. N. Romarión-Benitez V. (2017). [Comparative analysis about hospitalization characteristics in the mental health unit of a general acute care hospital]. *Vertex* 28 183–187. 29522623

[B42] Schrank B. Brownell T. Jakaite Z. Larkin C. Pesola F. Riches S. (2016). Evaluation of a positive psychotherapy group intervention for people with psychosis: pilot randomised controlled trial. *Epidemiol. Psychiatr. Sci.* 25 235–246. 10.1017/S2045796015000141 25698298 PMC6998731

[B43] Schwarzer R. Born A. (1997). Optimistic self-beliefs: assessment of general perceived self-efficacy in thirteen cultures. *World Psychol.* 3 177–190.

[B44] Shafir T. Orkibi H. Baker F. A. Gussak D. Kaimal G. (2020). Editorial: the state of the art in creative arts therapies. *Front. Psychol.* 11:68. 10.3389/fpsyg.2020.00068 32116898 PMC7012801

[B45] Sheikh A. A. Shaffer J. T. (1979). *The Potential of Fantasy and Imagination.* New York, NY: Brandon House.

[B46] Stefan G. Asnaani A. Vonk I. J. J. Sawyer A. T. Fang A. (2014). Erratum to: the efficacy of cognitive behavioral therapy: a review of meta-analyses. *Cogn. Ther. Res.* 38:368 10.1007/s10608-013-9595-3 PMC3584580 23459093

[B47] Teglbjaerg H. S. (2011). Art therapy may reduce psychopathology in schizophrenia by strengthening the patients’ sense of self: a qualitative extended case report. *Psychopathology* 44 314–318. 10.1159/000325025 21659793

[B48] Ting N. I. Bing-Shuang H. U. (2012). The application and development of art therapy for the past 10 years and its future in China. *J. Southwest Jiaotong Univ.* 013 92–97. 10.3969/j.issn.1009-4474.2012.03.019

[B49] Vaskinn A. Ventura J. Andreassen O. A. Melle I. Sundet K. (2015). A social path to functioning in schizophrenia: from social self-efficacy through negative symptoms to social functional capacity. *Psychiatry Res.* 228 803–807. 10.1016/j.psychres.2015.05.019 26089018

[B50] Wilkinson R. A. Chilton G. (2013). Positive art therapy: linking positive psychology to art therapy theory, practice, and research. *Art Therapy* 30 4–11. 10.1080/07421656.2013.757513

[B51] Zhang J. X. Schwarzer R. (1995). Measuring optimistic self-beliefs: a Chinese adaptation of the general self-efficacy scale. *Psychologia* 38 174–181. 10.1080/09515089508573160

